# Age-related differences in motor unit discharge rate modulation across contraction levels

**DOI:** 10.1007/s11357-026-02383-9

**Published:** 2026-06-30

**Authors:** Christopher D. Connelly, Tamara Valenčič, Haydn Thomason, Ed Sadula, Gregory E. P. Pearcey, Mathew Piasecki, Jonathan P. Folland, Jakob Škarabot

**Affiliations:** 1 School of Sport, Exercise and Health Sciences, Loughborough University, Loughborough, UK; 2 School of Human Kinetics and Recreation, Memorial University of Newfoundland, St. John’s, Canada; 3 Centre of Metabolism, Ageing & Physiology (COMAP), National Institute for Health Research (NIHR) Nottingham Biomedical Research Centre, School of Medicine, University of Nottingham, Derby, UK

**Keywords:** Age-related strength loss, Motoneuron, Persistent inward currents

## Abstract

Age-related strength loss may reflect morphological and neural adaptations that differ between muscles. However, it is unclear how ageing affects motor unit (MU) discharge rate modulation and estimates of persistent inward currents (PICs) across contraction levels. Thirty young (24 ± 4 years) and thirty older adults (73 ± 4 years) underwent magnetic resonance imaging and performed unilateral isometric dorsiflexion and knee extension contractions. Individual MU spike trains were identified from decomposed high-density electromyography recordings of tibialis anterior (TA) and vastus lateralis (VL) during triangular contractions at 30%, 50%, and 70% of maximal voluntary force (MVF). Older adults exhibited lower isometric strength in both tasks and smaller quadriceps but greater shank anterior compartment cross-sectional area compared to young. TA MU discharge rate was lower in older adults at all contraction levels but only at ≤ 50% MVF in VL. Discharge rate hysteresis (∆F), an estimate of PIC contribution to MU discharge, was lower in older adults at all TA contraction levels, but only at 70% MVF in VL, and in both muscles older adults exhibited an attenuated increase in ∆F across contraction levels. The ascending discharge rate non-linearity did not differ between groups, except at 30% MVF in VL where it was greater in older adults. Older adults also exhibited lower acceleration and attenuation slopes in TA, but not VL. These results reveal muscle-specific differences in MU discharge rate modulation with ageing, suggesting that the age-related strength loss is likely underpinned to a greater extent by morphological and neural factors in knee extensors and dorsiflexors, respectively.

**Supplementary Information:**

The online version contains supplementary material available at https://doi.org/10.1007/s11357-026-02383-9.

## Introduction

Adults experience a decline in muscle mass and muscle force generating capacity as they age, which results in a decline of physical function, increasing the risk of falls, hospitalisations, and numerous other adverse health outcomes [1, 2]. Individuals in the eighth decade generally undergo strength loss of ~ 3% per year but muscle mass only declines by ~ 1% per year, suggesting that factors other than the size of muscle tissue contribute to age-related loss of strength [3, 4].

Motor units (MU), which are comprised of an alpha motoneuron and all the muscle fibres it innervates, act as neuromechanical transducers in the central nervous system, transforming an activation signal into the mechanical output of muscle fibres [5]. Voluntary muscle forces are controlled by the recruitment of MUs and their discharge rate modulation, both of which might be influenced by the ageing process [6]. Indeed, meta-analysis has suggested that older adults exhibit lower MU discharge rates compared to young [7]. Notably, however, individual studies on this phenomenon exhibit heterogenous outcomes from low populations of MUs [8–10], examinations of MU discharge rates during low to moderate forces (< 50% of maximal voluntary force, MVF) [11], or using low sample sizes [12]. Examining the age-related differences in MU discharge rate at higher contraction forces is critical for understanding the age-related loss in strength as well as for activities of daily living; for example, a chair rise has been shown to require 80% of the maximum voluntary joint moment of older adults compared to 42% in young adults [13], with many activities of daily living also requiring larger contraction forces [14].

The age-related decline in strength does not appear to be uniform across muscles, with cross-sectional data indicating ~ 32% lower isometric knee extensor strength of older vs. young adults [11], whilst ankle dorsiflexor strength was only ~ 14% lower in older adults [15]. Similarly, the quadriceps appear to undergo significant atrophy in older compared to young adults unlike the anterior compartment of the shank which appears relatively well preserved with ageing [16]. However, it remains unknown to what extent age-related neural alterations differ between muscles. In young adults, the vastus lateralis (VL) and tibialis anterior (TA) muscles have markedly different MU properties, with the VL having a greater estimated innervation ratio compared to TA (~ 5990 vs. ~ 1845) [17] but much lower discharge rates during a maximum voluntary contraction (~ 25 pps vs. ~ 42 pps) [8, 18]. These intrinsic differences in MU organisation along with the discrepancy in the age-related decline of muscle mass and strength between muscles suggest that ageing may not affect MU properties uniformly.

Previous literature has suggested that the age-related differences in MU discharge rate may be greater at higher levels of contraction [7]; however, the mechanisms for these observations remain unclear. A possible age-related reduction in discharge rate, particularly at higher contraction levels, has been attributed to denervation and compensatory MU remodelling, resulting in fewer MUs with larger innervation ratios [19], but could also be due to alterations in synaptic input [20, 21] or the intrinsic excitability of motoneurons. The latter is facilitated by persistent inward currents (PICs), which are depolarising currents on motoneuron dendrites mediated by ligand-type Ca^2+^ and Na^+^ channels that result in acceleration and prolongation of MU discharge rate [22, 23]. The control of PICs is an active process of balancing facilitation and inhibition, as PICs are proportional to the neuromodulatory input from the brainstem raphe nuclei that diffusely project monoamines (serotonin, noradrenaline) onto motoneurons via long descending axons [22–24], and dampened via inhibitory synaptic input [25]. During low levels of contraction (≤ 30% MVF), the estimates of PIC contribution to self-sustained MU discharge rate (onset-offset hysteresis; ∆F [26]) have been shown to be reduced in older adults in a number of muscles including the tibialis anterior (TA) [27, 28], vastus lateralis (VL) [29], soleus [27, 28], and biceps and triceps brachii [30]. Although preliminary evidence suggests that remote facilitation of PICs is maintained, and inhibitory control is impaired in ageing adults [27], our understanding of age-related alterations of PIC contribution to MU discharge rate is limited to lower-threshold MUs examined during low contraction levels (≤ 30% MVF). This represents a key limitation, as higher contraction levels are associated with increased neuromodulation and greater reciprocal excitation-inhibition within the motoneuron pool, which experimental and in silico evidence suggests may disproportionately influence PIC contribution to MU discharge rate compared to lower levels of excitation and force production [31]. Consequently, age-related differences in neuromodulation and excitation-inhibition coupling may become more pronounced at higher contraction levels, potentially contributing to the greater reductions in MU discharge rate observed in older adults under these conditions, but this remains to be directly examined.

The aim of the present study was to compare the modulation of MU discharge rate and estimates of PICs across a spectrum of contraction levels in young and older adults. Furthermore, the aim was to compare whether any age-related differences in MU discharge rate and estimates of PICs are different between muscles. For the latter, we selected TA and VL muscles as they have previously been shown to exhibit different age-related declines in strength [11, 15] and MU and motor pool characteristics [8, 17, 18]. It was hypothesised that MU discharge rate and estimates of PICs would be lower in older adults, with these differences exacerbated at higher contraction levels and with a more pronounced difference in the VL that might explain the greater age-related loss in strength and size previously demonstrated in this muscle [11, 15, 19].

## Methods

### Participants

Sixty participants with low to moderate levels of physical activity were recruited for the study, including 30 young adults (15 females; mean ± SD, age: 24 ± 4 years, stature: 1.73 ± 0.10 m, mass: 67 ± 11 kg, body mass index: 22.4 ± 1.9 kg/m^2^) and 30 older adults (15 females, age: 73 ± 4 years, stature: 1.67 ± 0.10 m, mass: 73 ± 12 kg, body mass index: 26.4 ± 2.8 kg/m^2^). Older adults were shorter than young adults (*p* = 0.0216) but had greater mass (*p* = 0.0364) and body mass index (*p* < 0.0001). All participants were free from cardiovascular, neurological, or respiratory disorders, or musculoskeletal injury of the lower limbs. Participants were eligible for the young group if they were aged 18–35 years, and for the older group if they were aged over 65 years. Additionally, participants were ineligible if they were sedentary or engaged in systematic training or competition, including resistance training, endurance training and team sports. Written informed consent was obtained from all eligible participants. The study was approved by the Loughborough University Ethics Committee (2021-5361-4724), and all study procedures were performed in accordance with the *Declaration of Helsinki* except for registration in database.

All participants completed the International Physical Activity Questionnaire (IPAQ) [32] to estimate daily physical activity level, which was not different between groups (3059 ± 1696 vs. 3926 ± 2238 MET·min/week; *p* = 0.096). Older participants’ frailty status was determined by adapting the frailty phenotype guidelines of Fried et al. [33], using the following five criteria: (1) sarcopenia, determined by quadriceps peak cross-sectional area relative to young individuals (*z*-score ≤ −2) [19]; (2) exhaustion, determined via a questionnaire (answered yes ≥ 3–4 days per week to either: ‘I felt that everything I did was an effort’ or ‘I could not get going’); (3) slowness, determined by a 4 m walk test (≤ 0.8 m/s) [34]; (4) weakness, determined by grip strength test (in the lowest 20% by sex and BMI; Jamar Hydraulic Hand Dynamometer, set to position two); and (5) physical activity, determined via IPAQ (estimated expenditure for males < 383 kcals per week, or estimated expenditure for females < 270 kcals per week). Participants were considered robust if no criteria were met, pre-frail if 1–2 criteria were met, and frail if ≥ 3 criteria were met. Out of 30 participants, we found that none of the older adults were classified as frail, 18 were pre-frail and 10 were robust (*n.b.* in two individuals, calculation was not possible due to no imaging data; for details see below).

### Study protocol

Participants visited the laboratory on four occasions: a familiarisation session, a dorsiflexion testing session, a knee extension testing session, and a magnetic resonance imaging (MRI) scan. In the familiarisation session, participants completed the same tasks as in the testing sessions to become accustomed to the maximal contractions and force-matching tasks during unilateral isometric dorsiflexion and knee extension with their dominant leg. For each session participants were instructed to arrive well rested, having refrained from strenuous activity the day prior, and to avoid caffeine on the day of the testing sessions.

#### MRI

T1-weighted axial MR images were taken from the twelfth thoracic vertebrae to the calcaneus of both legs with a 3 T scanner (Discovery MR750w; GE Healthcare, Chicago, IL, USA). Images were acquired in five overlapping blocks (time of repetition 600 ms, time of echo 8 ms, field of view 450 × 450 mm, image matrix 320 × 320, pixel size 1.4 × 1.4 mm, slice thickness 5 mm, interslice gap 5 mm). Participants lay supine, with the ankle at 90˚, the knee and hip fully extended, and the arms folded across the chest in a whole-body coil. Oil-filled capsules were placed laterally on the right leg at equidistant intervals to facilitate alignment of the overlapping blocks during analysis.

Two older and one young adult were unable to undergo MRI due to contraindications. Additionally, one young individual’s scan was excluded from quadriceps analysis due to an artefact in the images. Therefore, the results of MRI analysis are based on *n* = 57 for anterior compartment of the shank and *n* = 56 for quadriceps femoris. Additionally, for calculation of frailty phenotype (see ‘Participants’ section) the sample size was *n* = 28.

### Force measurements

All force measurements were made with two rigid custom-built isometric dynamometers, with straps tightly fastened over the pelvis and shoulders. Dorsiflexion force recordings were taken with the participants seated with 120° of hip flexion and the knee fully extended (180°) in a custom-built chair fitted with an ankle dynamometer (NEG1, OT Bioelettronica, Torino, Italy) with the ankle at 10° plantarflexion (0° = neutral). The knee joint was strapped down to minimise any knee flexion during dorsiflexion. The foot was secured to a rotating wooden footplate, with proximal (over the dorsum of the foot) and distal (on the distal third of the metatarsals) foot straps and with the heel supported by a small cup. The ankle joint centre was aligned with the axis of rotation of the footplate. The footplate was fixed in place by a strain gauge (CCT Transducer s.a.s., Torino, Italy) arranged in series. The analogue force signal was amplified (× 200; Forza-B, OT Bioelettronica, Torino, Italy) and sampled at 2048 Hz using an analogue-to-digital (A/D) converter (Micro 1401–4; CED Ltd., Cambridge, UK).

Knee extension recordings were performed with participants seated with the knee and hip flexed at 115° and 125°, respectively (180° = full extension). Force recordings during knee extension were measured with a calibrated S-beam strain gauge (linear range 0–1.5 kN; Force Logic, Swallowfield, UK). During knee extension the strain gauge was attached perpendicular and posterior to the tibia with a strap (35 mm reinforced canvas webbing) placed ~ 3–4 cm above the medial malleolus. The analogue force signal was amplified (× 370) and sampled at 2048 Hz using an A/D converter (Micro 1401-4; CED Ltd., Cambridge, UK).

The force signal during both knee extension and dorsiflexion was acquired in Spike2 software (version 10; CED Ltd., Cambridge, UK). Force was also concurrently sampled (2048 Hz) and recorded in OTBiolab + software (Quattrocento; OT Bioelettronica, Torino, Italy) using a multichannel amplifier (16-bit, Quattrocento; OT Bioelettronica, Torino, Italy) for synchronisation with high-density surface electromyography (HDsEMG) recordings. Visual force biofeedback was provided during all experimental procedures on a monitor ~ 1 m in front of participants.

### Electromyography recordings

Each testing session began with identification of the motor point on the VL for knee extension and TA for dorsiflexion. The motor point was identified as the site on the muscle that produced the greatest twitch in response to low intensity percutaneous electrical stimulation (maximum output 400 V, 0.2 ms pulse width; DS7R; Digitimer, Welwyn Garden City, UK).

Preparation of the skin over the VL and TA involved shaving and abrasion. After that, 64-channel HDsEMG electrode grids (13 × 5, 1 mm electrode diameter, 8 mm interelectrode distance; OT Bioelettronica) were covered with a bi-adhesive foam layer (Spes Medica, Battipaglia, Italy) with the cavities filled with conductive paste (Ten20, Weaver and Co., Aurora, CO, USA). The HDsEMG electrodes were placed on the muscle bellies aligned with the presumed muscle fibre orientation. The VL electrode grid was placed distal of the motor point at 30° from a line drawn between the anterior superior iliac spine and the lateral patella [35]. The TA electrode grid was placed distal to the motor point over the belly of the TA muscle. Reference electrodes were placed over the fibular head for knee extension and over the medial malleolus for dorsiflexion, with a dampened strap around the non-dominant ankle used to ground the signal. HDsEMG signals were recorded in a monopolar configuration, bandpass filtered (10–500 Hz), sampled at 2048 Hz using a 16-bit multichannel amplifier (Quattrocento; OT Bioelettronica, Torino, Italy), and acquired using OT Biolab + software (OT Bioelettronica, Torino, Italy).

### Experimental procedures

Identical dorsiflexion and knee extension tasks were completed. Participants began with a brief warm up consisting of 3 × 50%, 3 × 75% and 1 × 90% of perceived maximum effort contractions, each lasting ~ 3 s and separated by 15–30 s rest. After that, four contractions with maximal effort were performed separated by ≥ 30 s rest, in which participants were asked to perform the contractions “as hard as possible” for 3–5 s. During the maximal effort contractions, participants received strong verbal encouragement and visual force biofeedback was provided. Participants were instructed to try and beat their previous highest score (indicated by a horizontal cursor on the monitor) during successive efforts. The MVF was taken as the highest instantaneous force achieved during any contractions with maximal effort.

Participants then completed several submaximal contractions in which they were asked to follow a force target as closely as possible. Specifically, participants were tasked to perform triangular-shaped ramp contractions by following a target trace with a 10 s linear ascending and then 10 s linear descending slope, peaking at 15, 30, 50, and 70% MVF. Two contractions were performed at each force level and separated by 30–60-s rest.

### Data analysis

Offline analyses were performed using MATLAB (R2021b; Mathworks Inc., Natick, MA, USA).

#### Force signal

The force signal was filtered using a fourth-order, zero-lag low-pass Butterworth filter with a cut-off frequency of 20 Hz and gravity corrected.

#### HDsEMG analysis

High-density surface EMG signals were inspected, and poor-quality channels were removed based on their amplitude and area under the power spectrum using a semi-automated tool in MATLAB. The signals were then decomposed using the Convolution Kernel Compensation algorithm to obtain the individual MU spike trains [36]. This algorithm uses blind source separation principles, inverting the EMG mixing model to estimate the MU filter, which represents weights of the linear spatial-temporal combination of HDsEMG channels and generates an estimation of each individual MU spike train [37, 38]. Decomposed signals at the same force level were then concatenated and MU filters transferred to enhance MU yield [39, 40] followed by manual inspection and editing by trained operators as previously described [41]. Any duplicate MU spike trains that were detected as sharing 30% of the same MU discharges (discharge tolerance of 0.5 ms, i.e. 1 sample) were deleted based on lower estimated decomposition accuracy assessed by pulse-to-noise ratio. Motor unit spike trains were kept for further analysis if they had a pulse-to-noise ratio of 30 dB or greater and a consistent discharge pattern throughout the contraction [42].

#### Motor unit tracking

**Fig. 1:**
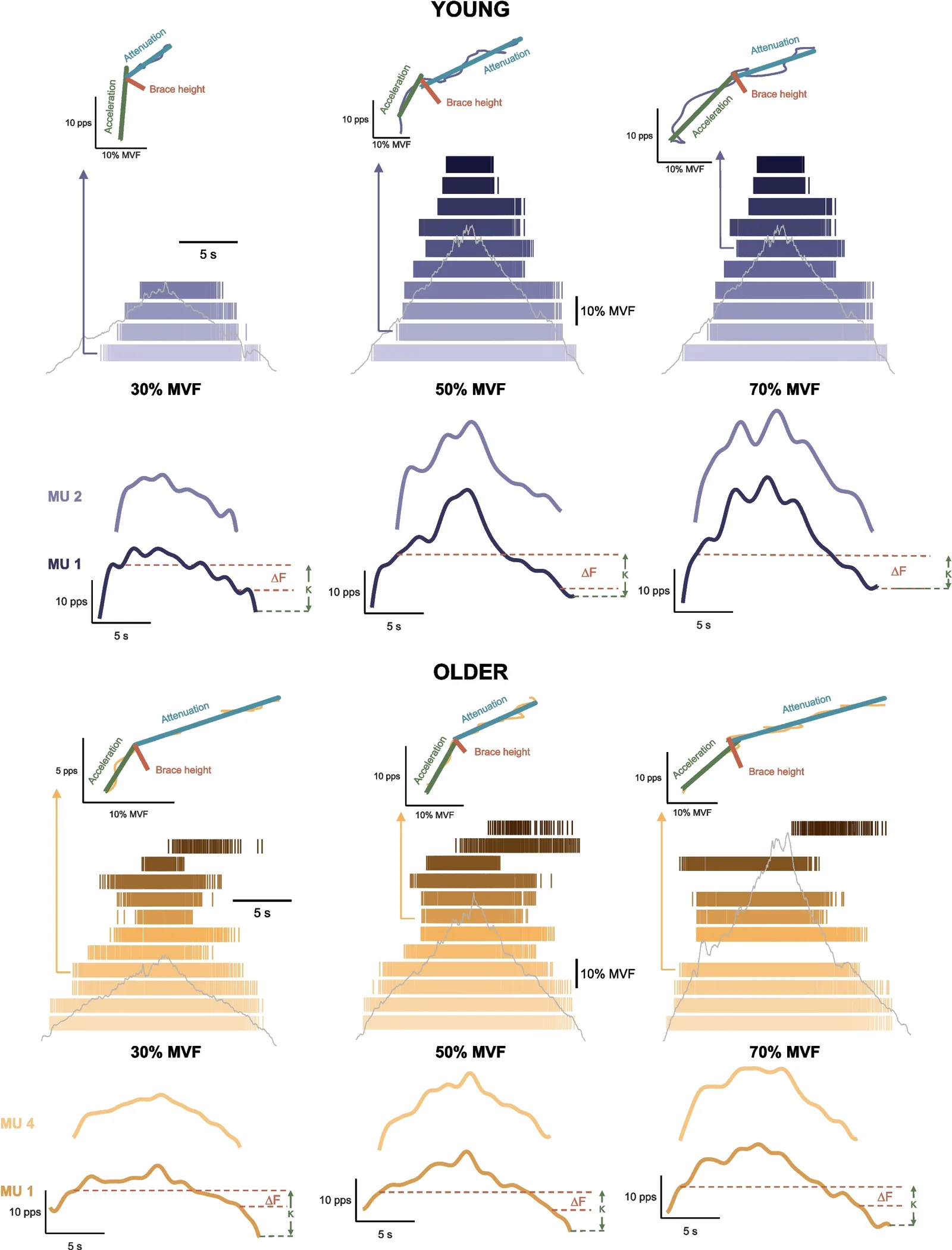
Examples of motor unit discharge behaviour across contraction levels in young and older adults. For each group, i.e. young (upper panels, blue) and older adults (lower panels, orange) a raster plot of *tracked* motor unit (MU) discharges is shown in the centre during triangular contractions to 30, 50, and 70% of maximal voluntary force (MVF). In the upper tier of each group’s panel block, the ascending discharge rate of representative units expressed as a function of force is shown, along with the calculated brace height, and the acceleration and attenuation slopes. In the lower tier of each group’s panel block a representative pair of smoothed MU discharge rates is shown, with onset-offset hysteresis (∆F) annotated for each test unit, and with κ representing the maximal discharge rate modulation of the control unit between test unit recruitment and control unit derecruitment, to which ∆F were normalised

The discharge patterns of MUs were analysed independently across contraction force levels (see supplementary material) and with MU tracking to account for the potential replicates across contraction levels. A similar procedure to that described by Škarabot et al. [31] was used to track MU spike trains across contraction levels. Briefly, separation vectors (MU filters) were used to detect spike trains of MUs that were identified across multiple contraction levels. Specifically, the decomposition results from each contraction level were concatenated, and the MU filters were applied from signals recorded during lower to those recorded during higher contraction levels to identify common MU spike trains. Contractions at 15% MVF were excluded from this part of analysis, as it was unlikely any MUs could be tracked from 15 to 70% MVF due to the inherent sampling bias of HDsEMG decomposition (i.e. difficulty in identifying low-threshold MUs in the presence of higher-threshold MUs with large potentials) [37]. As part of the tracking process, duplicate MU spike trains were deleted using the same criteria as above (see HDsEMG analysis). Tracking low threshold MUs whilst high threshold MUs are concurrently active can be challenging [37], and therefore, we tracked MUs from neighbouring contraction levels 30–50% MVF and 50–70% MVF as well as 30–50–70% MVF to enhance MU yield (Fig. 1). The results were found to be similar with or without tracking of MUs across contraction intensities; therefore, we chose to principally present results of tracked MUs, whereas the results of non-tracked MUs are located in the Supplementary Material.

#### Motor unit discharge properties

For all MU discharge-related outcome variables, MU spike trains from triangular ramp contractions were smoothed with support vector regression to produce a continuous estimate of MU discharge patterns using parameters as previously described [43]. Peak discharge rate was calculated as the peak of the smoothed discharge rate. The relative force produced at the time a given MU was recruited (i.e. the first spike in the spike train was identified) was considered the recruitment threshold.

#### Analysis of motor unit discharge patterns

A hallmark of PIC-mediated actions on motoneurons is the prolonged effect of synaptic input. In cases of linearly increasing and decreasing excitatory synaptic input, the prolongation of MU discharge is evident as the discharge onset-offset hysteresis, whereby a motoneuron is derecruited at lower levels of synaptic input than was required for its recruitment [44]. In human recordings, onset-offset hysteresis can be quantified as the difference in discharge rate of a lower-threshold (control) unit at the instants of higher-threshold (test) unit recruitment and derecruitment (ΔF) [26, 45], which we calculated in the present study. In the case of non-tracked MUs, we used a ‘unit-wise’ approach which provides a single, mean ΔF value for a given test unit which may be paired with multiple control units [30, 46]. However, in the case of tracked MUs, the calculations of ΔF were made for individual pairs (Fig. 1) in order not to bias the estimation of hysteresis (e.g. to avoid a potential case of different number of tracked test units being deemed suitable at different contraction levels due to subtly meeting the inclusion criteria). A pair of MUs was deemed suitable for calculation of ΔF if (1) the test unit was recruited > 1 s after the control unit, to ensure full activation of control unit PICs [47], (2) the control unit discharge rate was > 0.5 pps whilst the test unit was active, to reduce the influence of MU saturation on ΔF calculation [48], and (3) the rate-rate correlation of pairs of MUs was *r*^2^ > 0.7 [49], to increase the likelihood that the MU pair shared common input. To account for potential age-related differences in descending MU discharge rate modulation of control units and/or across contraction levels, we additionally normalised ΔF values to the maximal discharge rate modulation for each test-control unit pair (i.e. ΔF/*κ*, where *κ* represents the difference in control unit discharge rate between test unit recruitment and control unit derecruitment, Fig. 1) [31].

To further appreciate the contribution of PICs to MU discharge rate patterns, we examined the ascending MU discharge modulation via quasi-geometric analysis. Specifically, we quantified the non-linearity of the ascending MU discharge rate (termed ‘brace height’) to disentangle the relative contribution of neuromodulation and excitation-inhibition patterns to MU discharge [50]. That is, utilising in silico studies with biologically realistic motoneuron pools, it has been shown that the ascending discharge rate non-linearity is associated with neuromodulation, whereas onset–offset hysteresis is dependent on both neuromodulation and the pattern of inhibitory synaptic input relative to excitation [31, 50, 51]. For this analysis, the smoothed discharge rate of any given MU was expressed as a function of force; from there, the relative deviation from linearity (brace height) was quantified as the maximum orthogonal vector between the smoothed discharge rate and the theoretical linear discharge (a linear line between onset and peak firing; Fig. 1). To minimise the confounding effects of the absolute ascending discharge rate modulation (e.g. when comparing individuals of different ages that are likely to exhibit different peak discharge rate, or when examining different contraction levels), the brace height values were normalised to a right triangle with a hypotenuse between onset and peak discharge rate.

The quantification of brace height provides additional metrics which may elucidate a deeper insight of the control of MU discharge behaviour. Specifically, we measured the slope of the initial acceleration phase of MU discharge and the following attenuation phase (Fig. 1), which were segmented at the point of brace height occurrence [50]. The acceleration phase corresponds to the secondary range of MU discharge and features an increase in MU discharge rate, thought to be due to PIC amplification [52]. The subsequent attenuation phase corresponds to the tertiary range of MU discharge, which is likely when PICs are fully activated, meaning that further increases in synaptic input result in an attenuated increase in discharge rate [53]. In addition to brace height and ΔF metrics, these variables were quantified to potentially allow us to decouple the influence of neuromodulation and pattern of inhibition to motoneurons. Specifically, in simulations with a fixed excitation level, the attenuation slope has been shown to be sensitive to the pattern of inhibition whereas the acceleration slope may be more sensitive to the amount of neuromodulation and the total inhibitory input [31, 50].

Motor units were excluded from the quasi-geometric analysis if they had a negative slope during the acceleration phase, a normalised brace height greater than 200%, or if peak MU discharge rate was reached after peak force.

#### MRI

The MR images of the dominant leg were analysed for the anterior compartment of the shank (tibialis anterior, extensor digitorum longus and extensor hallucis longus) which was segmented as one whole and quadriceps (rectus femoris, vastus lateralis, vastus medialis, and vastus intermedius) in which each constituent quadricep was segmented individually and then summed. This analysis was performed using an open access DICOM image-analysis software (HOROS, version 2.2.0, www.thehorosproject.org). From the axial images, the image with the subjectively largest anatomical cross-sectional area (ACSA) and two slices either side (proximal and distal) were first manually segmented for each muscle/compartment, with additional slices segmented according to the ACSA values to ensure that at least two slices were segmented either side of the largest ACSA. A cubic spline function was fit to these five ACSAs generating 1000 interpolated values (R2021b; Mathworks Inc., Natick, MA, USA). The peak ACSA was defined as the maximum interpolated value. In the quadriceps, the sum of each constituent muscle peak ACSA was defined as maximum quadriceps ACSA.

### Statistical analysis

All statistical analysis was performed in R (RStudio, v 1.4.1106, R Foundation for Statistical Computing, Vienna, Austria).

To assess age-related differences in MU discharge characteristics, linear mixed effects models (*lme4* package; ver. 1.1.35.3, [54]) were constructed with age group (young vs. older), muscle (TA, VL), contraction level (30, 50, and 70% MVF) and their interaction as fixed factors with participant identifier and MU identifier as random intercepts, which were nested in a hierarchical structure. We also included MU recruitment threshold as a covariate to control for its influence on MU discharge behaviour [55]. Additionally, although sex differences were not the focus of this study, to account for potential differences between males and females [46], sex was used as a covariate in the statistical model. For example, the Wilkinson notation for the model with peak discharge rate as the outcome variable was as follows: Peak discharge rate ~ Age Group × Muscle × Contraction Level + Recruitment Threshold + Sex + [1 | Participant ID] + [1 | Participant ID | MU ID]. For ΔF, in order to account for the influence of both control and test units the model was as follows: ΔF ~ Age Group × Muscle × Contraction Level + Test Recruitment Threshold + Control Recruitment Threshold + Sex + [1 | Participant ID] + [1 | Participant ID | Test MU ID] + [1 | Participant ID | Test MU ID]. Quantile–quantile plots and histograms were used to verify the assumptions of normality and linearity of residuals. Observations with studentised residuals exceeding ± 3 were identified as outliers and excluded from the models. Square root (sqrt) and log transformation of dependent variables were used when residuals were found to be non-normally distributed. For variables containing negative values, a constant was added, which was the smallest whole number required to shift all values into the positive range before transformation. The selection of transformation function was determined based on visual inspection of residuals (as described above) and on model fit using Akaike Information Criterion and Bayesian Information Criterion. The following variables were transformed: peak discharge rate (log), ΔF (sqrt + 8), brace height (log), acceleration slope (sqrt), and attenuation slope (log + 1).

Similarly, linear mixed effects models were used to assess if there were differences in MVF due to factors of age group and joint action (knee extension and dorsiflexion), and to assess differences in muscle peak ACSA due to age group and muscle group (quadriceps and anterior compartment), with participant identifier as a random intercept and sex as covariate in both instances. Both MVF and peak ACSA underwent log transformation. Linear models were used to assess differences in IPAQ score, MU counts and participant characteristics (height, body mass and body mass index).

The significance of the model fixed effects and interactions was assessed using Type II ANOVA based on Wald chi-square tests from the *car* package (ver. 3.1.3). Significant main effects and interactions were further investigated using simple effects, interaction contrasts and pairwise comparisons of estimated marginal means (*emmeans* package; ver. 1.11.1, [56]) with Bonferroni-adjusted p-values to control for multiple comparisons. Cohen’s *d*, calculated as the difference in estimated marginal means divided by the residual standard deviation of the linear mixed effects model, was used to estimate effect sizes of post hoc comparisons. Significance was set at an alpha level of 0.05. Data are presented as estimated marginal means [95% confidence interval].

## Results

### Maximum isometric strength and muscle size

We first quantified age-related differences in maximal voluntary isometric strength and peak ACSA for the two muscle groups of interest to establish the functional and morphological basis for subsequent MU analyses, respectively. For MVF there were main effects of age group (*χ*^2^(1) = 55.0, *p* < 0.0001), joint action (*χ*^2^(1) = 365.0, *p* < 0.0001) and an age group × joint action interaction (*χ*^2^(1) = 48.9, *p* < 0.0001). Post hoc comparisons showed that compared to young adults, older adults had a lower dorsiflexion MVF (261 [231, 291] N vs. 307 [276, 337] N; *p* = 0.0364; *d* = 0.68) and lower knee extension MVF (410 [379, 440] N vs. 627 [597, 657] N; *p* < 0.0001; *d* = 3.23), but the age-related difference was greater in the latter than the former (*p* < 0.0001; *d* = 2.55).

For peak ACSA there were main effects of age group (*χ*^2^(1) = 18.2, *p* < 0.0001), muscle (*χ*^2^(1) = 9484.2, *p* < 0.0001) and an age group × muscle interaction (*χ*^2^(1) = 137.7, *p* < 0.0001). Older adults had a ≈10% larger peak ACSA of the shank anterior compartment compared to young adults (*p* = 0.0208; *d* = 0.86), but a ≈30% lower total quadriceps femoris peak ACSA (*p* < 0.0001; *d* = 3.57; Table 1).

**Table 1:** Age-related differences in anatomical peak cross-sectional area of shank anterior compartment and quadriceps femoris

Muscle group	Anatomical peak cross-sectional area (cm^2^)	
	**Young**	**Older**
**Anterior compartment**	9.4 [9.0, 9.9]	10.3* [9.8, 10.8]
**Quadriceps femoris**	75.4 [71.6, 79.4]	52.6*** [49.9, 55.4]

Quadriceps femoris is the sum of rectus femoris, vastus medialis, vastus intermedius and vastus lateralis. ***p < 0.001 and *p < 0.05 denote difference compared to young. Data are presented as estimated marginal mean [95% confidence interval]

### Motor unit discharge rate and hysteresis

To assess age-related differences in motor unit discharge properties and the contribution of PICs to self-sustained discharge rate, we compared peak MU discharge rate and discharge rate hysteresis (∆F) of the identified MUs between young and older adults across muscle groups of interest and different contraction levels. There was no between-group difference in the number of unique MUs identified in TA; older adults had a total of 474 compared to 388 in young (mean ± SD per participant: 16 ± 7 vs. 13 ± 8; *p* = 0.1507). However, in VL, older adults had a greater number of unique MUs identified compared to young adults, with 394 compared to 179 (mean ± SD per participant: 13 ± 11 vs. 6 ± 5; *p* = 0.0017; Supplemental Table 1).

**Fig. 2:**
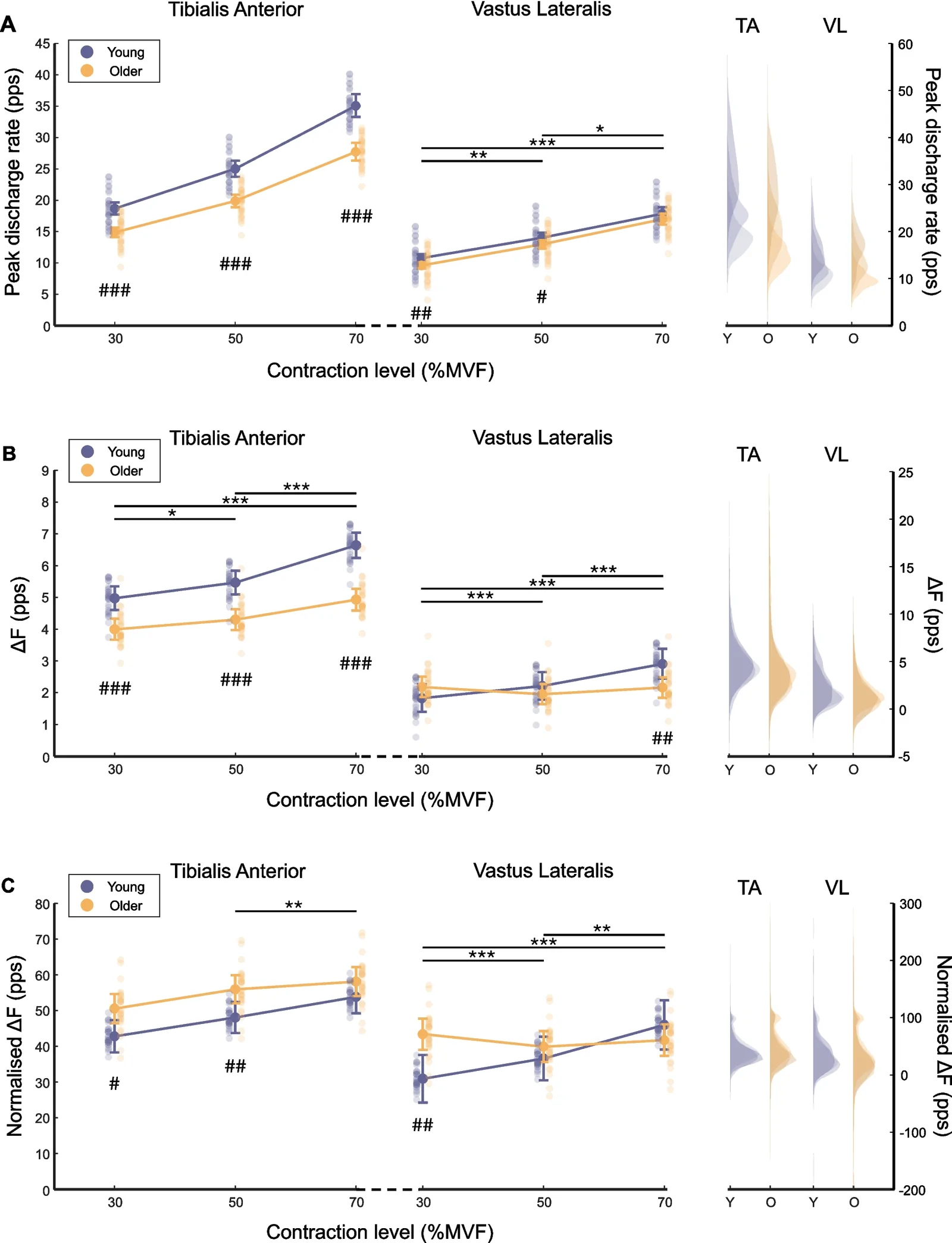
Motor unit discharge rate and hysteresis in tibialis anterior and vastus lateralis of young and older adults by contraction level. **A** Peak discharge rate (pulses per second, pps). **B** Onset-offset discharge hysteresis of MU pairs (ΔF). **C** ΔF normalised to the difference between control unit discharge rate at test unit recruitment and at control unit derecruitment. Blue (young) and orange (older) circles represent estimated marginal means from the linear mixed effects statistical model, with the error bars representing 95% confidence intervals. Transparent circles represent individual participant model adjusted means for the whole statistical model (i.e. not by muscle) and kernel density plots display the distribution of all MU data points. ^###^*p* < 0.001, ^###^*p* < 0.01, and ^#^*p* < 0.05 denote between-group differences at each contraction level; ^***^p < 0.001, ^**^p < 0.01, and ^*^p < 0.05 denote interaction contrast between groups across contraction levels. TA = tibialis anterior, VL = vastus lateralis, MVF = maximal voluntary force

Peak discharge rate during triangular contractions showed main effects of age group (*χ*^2^(1) = 26.0, *p* < 0.0001), muscle (*χ*^2^(1) = 3146.5, *p* < 0.0001) and contraction level (*χ*^2^(2) = 24213.3, *p* < 0.0001; Fig. 2A). There were also interactions of age group × muscle (*χ*^2^(1) = 63.8, *p* < 0.0001), muscle × contraction level (*χ*^2^(2) = 137.5, *p* < 0.0001), age group × muscle × contraction level (*χ*^2^(2) = 17.9, *p* = 0.0001) but not for age group × contraction level (*χ*^2^(2) = 5.4, *p* = 0.0662). In both muscles and both groups, with increasing contraction level peak discharge rate increased (all *p* < 0.0001; all *d* > 2.37). Pairwise comparisons between groups showed that young adults had a greater discharge rate than older adults at each contraction level in TA (all *p* < 0.0001; all *d* ≥ 2.24), and in VL at 30% (*p* = 0.0033; *d* = 1.13) and 50% (*p* = 0.0407; *d* = 0.77), but not at 70% MVF (*p* = 0.1864; *d* = 0.50). Significant interaction contrasts in VL also demonstrated that discharge rate in older adults increased to a greater extent than young from 30 to 50% (*p* = 0.0030; *d* = 0.36), 30 to 70% (*p* < 0.0001; *d* = 0.63) and from 50 to 70% MVF (*p* = 0.0401; *d* = 0.27). Conversely, in TA, there were no significant interaction contrasts between groups across contraction levels (all *p* = 1.0000, *d* ≤ 0.07).

The MU discharge rate hysteresis (ΔF) showed main effects of age group (*χ*^2^(1) = 13.8, *p* = 0.0002), muscle (*χ*^2^(1) = 387.3, *p* < 0.0001) and contraction level (*χ*^2^(2) = 719.9, *p* < 0.0001; Fig. 2B). There were also interactions of age group × muscle (*χ*^2^(1) = 12.0, *p* = 0.0005), age group × contraction level (*χ*^2^(2) = 135.8, *p* < 0.0001), muscle × contraction level (*χ*^2^(2) = 226.4, *p* < 0.0001) and age group × muscle × contraction level (*χ*^2^(2) = 16.6, *p* = 0.0002). In TA, pairwise between-group comparisons revealed that young adults had greater ΔF than older adults at all contraction levels (30%, *p* = 0.0002, *d* = 0.80; 50% and 70%, *p* < 0.0001 for both, *d* ≥ 0.94). In the VL, there was no difference in ΔF between groups at 30% (*p* = 0.1987; *d* = 0.32) or 50% (*p* = 0.3462; *d* = 0.23) but at 70% MVF young had greater ΔF than older adults (*p* = 0.0098; *d* = 0.66). In both TA and VL across all contraction levels, the modulation of ΔF was greater in young than older adults with increasing contraction force (TA: 30–50%, *p* = 0.0140, *d* = 0.14; 30–70%, *p* < 0.0001, *d* = 0.53 and 50–70%, *p* < 0.0001, *d* = 0.39; VL: 30–50%, 30–70% and 50–70%, all *p* < 0.0001, *d*: 30%–50% = 0.56, 30%–70% = 0.99, 50%–70% = 0.43). Within groups, in TA, ΔF increased with greater contraction level for both groups (all *p* < 0.0001, all *d* ≥ 0.25). In VL, young adults’ ΔF increased with increasing contraction level (all *p* < 0.001, all *d* ≥ 0.35), however, in older adults ΔF decreased from 30 to 50% MVF (*p* < 0.0001; *d* = 0.21) and increased from 50 to 70% MVF (*p* < 0.0001; *d* = 0.19), but ΔF was not different between 30 and 70% MVF (*p* = 1.0000; *d* = 0.02).

Normalised ΔF had main effects of age group (*χ*^2^(1) = 6.2, *p* = 0.0128), muscle (*χ*^2^(1) = 51.9, *p* < 0.0001) and contraction level (*χ*^2^(2) = 67.0, *p* < 0.0001; Fig. 2C). There were also interactions of age group × contraction level (*χ*^2^(2) = 23.8, *p* < 0.0001), muscle × contraction level (*χ*^2^(2) = 54.2, *p* < 0.0001) and age group × muscle × contraction level (*χ*^2^(2) = 16.6, *p* = 0.0002) but not for age group × muscle (*χ*^2^(1) = 0.5, *p* = 0.4838). In TA, older adults had a greater normalised ΔF at 30% and 50% but not at 70% MVF (30%, *p* = 0.0119, *d* = 0.29; 50%, *p* = 0.0085, *d* = 0.30; 70%, *p* = 0.1629, *d* = 0.16). In VL, older adults had a greater normalised ΔF at 30% (*p* = 0.0021; *d* = 0.47), but not at 50% (*p* = 0.3772; *d* = 0.13) or 70% MVF (*p* = 0.2990; *d* = 0.16). Additionally, interaction contrasts revealed that in TA normalised ΔF of young adults increased relative to older adults from 50 to 70% MVF (*p* = 0.0087; *d* = 0.14), and in VL the same was noted across all contraction levels (30 to 50%, 30 to 70%, and 50 to 70% MVF, all *p* ≤ 0.0011; all *d* ≥ 0.29). In TA, both groups normalised ΔF increased with each contraction level (all *p* ≤ 0.0347; all *d* ≥ 0.08). In VL, young adults normalised ΔF increased from 30 to 70% and 50 to 70% (both *p* < 0.0001; both *d* ≥ 0.35) but not from 30 to 50% MVF (*p* = 0.1510; *d* = 0.21). In VL of older adults, a decrease in normalised ΔF was noted from 30 to 50% (*p* = 0.0001; *d* = 0.13), with no differences noted from 30 to 70% (*p* = 0.8795; *d* = 0.06) or 50 to 70% MVF (*p* = 0.0940; *d* = 0.07).

*Summary*. Peak MU discharge rate was lower in older than young adults at all contraction levels in TA but only at ≤ 50% MVF in VL. Discharge rate hysteresis (ΔF) was lower in older adults at all TA contraction levels but only at 70% MVF in VL, with the increase across contraction levels attenuated in both muscles. When normalised to the maximum theoretical hysteresis, ΔF was instead greater in older than young adults at lower contraction levels in both muscles.

### The ascending motor unit discharge pattern modulation

**Fig. 3:**
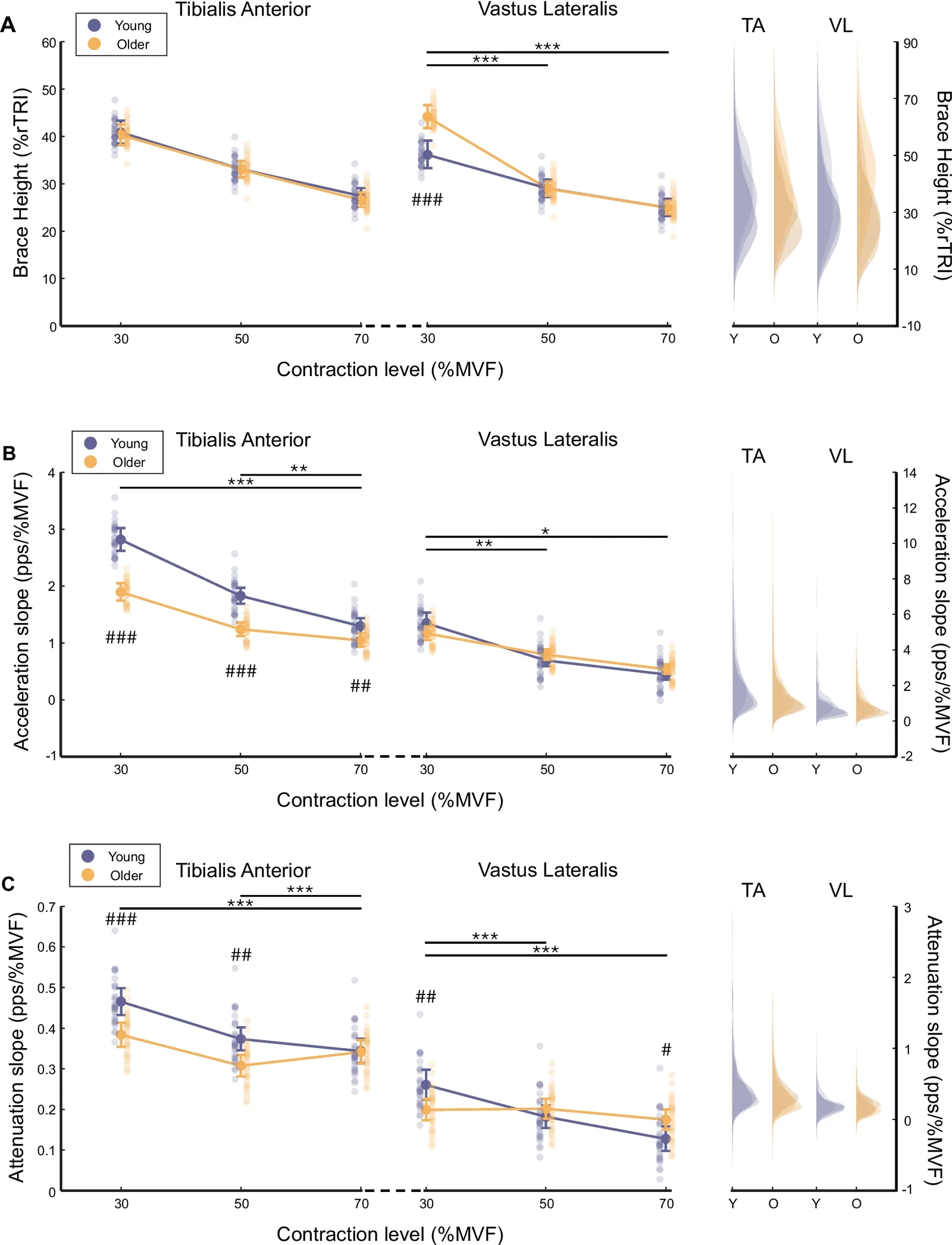
Motor unit discharge patterns in tibialis anterior and vastus lateralis of young and older adults by contraction level. **A** The non-linearity of the ascending discharge rate (brace height, expressed as a percentage of right triangle, %rTRI). **B** Acceleration slope of the ascending discharge (secondary discharge range; in pulses per second relative to maximal voluntary force, MVF). **C** Attenuation slope of the ascending discharge rate (tertiary discharge range). Blue (young) and orange (older) circles represent estimated marginal means from the linear mixed effects statistical model, with the error bars representing 95% confidence intervals. Transparent circles represent individual participant model adjusted means for the whole statistical model (i.e. not by muscle) and kernel density plots display the distribution of all MU data points. ^###^*p* < 0.001, ^##^*p* < 0.01, and ^#^*p* < 0.05 denote between-group differences at each contraction level; ^***^p < 0.001, ^**^p < 0.01, and ^*^p < 0.05 denote interaction contrast between groups across contraction levels. TA = tibialis anterior, VL = vastus lateralis, MVF = maximal voluntary force

To further appreciate the putative contribution of PICs to MU discharge characteristics, and to estimate the relative contribution of neuromodulation and inhibitory patterns to the observed age-related differences in MU discharge rates across muscle groups and contraction levels, we examined the ascending MU discharge patterns using quasi-geometric analyses including brace height, as well as acceleration and attenuation slopes. The ascending discharge rate non-linearity (brace height) showed no main effect of age group (*χ*^2^(1) = 0.1, *p* = 0.7637), but main effects were noted for muscle (*χ*^2^(1) = 20.4, *p* < 0.0001) and contraction level (*χ*^2^(2) = 663.6, *p* < 0.0001; Fig. 3A). Interaction effects were noted for each of age group × muscle (*χ*2(1) = 4.3, *p* = 0.0380), age group × contraction level (*χ*^2^(2) = 7.6, *p* = 0.0222), muscle × contraction level (*χ*^2^(2) = 26.5, *p* < 0.0001), and age group × muscle × contraction level (*χ*^2^(2) = 13.0, *p* = 0.0015). In either muscle, the only between-group difference noted was at 30% MVF in the VL where older adults had a greater brace height than young (*p* = 0.0001; *d* = 0.59). In VL, significant interaction contrasts also revealed that brace height of older adults decreased to a greater extent from 30 to 50% (*p* = 0.0001; *d* = 0.60) and from 30 to 70% MVF compared to young adults (*p* = 0.0006; *d* = 0.59). In both groups and muscles, with each increase in contraction level brace height decreased (all *p* ≤ 0.001; all *d* ≥ 0.44).

For the acceleration slope, there were main effects of age group (*χ*^2^(1) = 17.5, *p* < 0.0001), muscle (*χ*^2^(1) = 537.5, *p* < 0.0001) and contraction level (*χ*^2^(2) = 583.3, *p* < 0.0001; Fig. 3B). There were also interaction effects for age group × muscle (*χ*^2^(1) = 58.9, *p* < 0.0001) and age group × contraction level (*χ*^2^(2) = 25.9, *p* < 0.0001) but not for muscle × contraction level (*χ*^2^(2) = 2.7, *p* = 0.2627) or age group × muscle × contraction level (*χ*^2^(2) = 4.6, *p* = 0.1010). In TA, the acceleration slope was greater in young than older adults at all contraction levels (30% and 50%, both *p* < 0.0001, both *d* ≥ 0.74; and 70%, *p* = 0.0053, *d* = 0.36), but in the VL there were no between-group differences at any contraction level (all p ≥ 0.1115; all *d* ≤ 0.19). In TA, young adults acceleration slope decreased to a greater extent than older adults from 30 to 70% (*p* < 0.0001; *d* = 0.57) and from 50 to 70% MVF (*p* = 0.0039; *d* = 0.38), and in the VL similar significant interaction contrasts were noted (from 30 to 50%, *p* = 0.0075, *d* = 0.43; and 30 to 70% MVF, *p* = 0.019, *d* = 0.44). In both groups and muscles acceleration slope decreased with each increase in contraction level (all comparisons yielded *p* ≤ 0.0001, *d* ≥ 0.49, except for older TA from 50 to 70% MVF, *p* = 0.0137, *d* = 0.28).

Attenuation slope showed no main effects of age group (*χ*^2^(1) = 2.6, *p* = 0.1090) but significant main effects were noted for muscle (*χ*^2^(1) = 725.6, *p* < 0.0001) and contraction level (*χ*^2^(2) = 94.4, *p* < 0.0001; Fig. 3C). Significant interactions were observed for age group × muscle (*χ*^2^(1) = 18.2, *p* < 0.0001), age group × contraction level (*χ*^2^(2) = 38.9, *p* < 0.0001), muscle × contraction level (*χ*^2^(2) = 24.4, *p* < 0.0001) and age group × muscle × contraction level (*χ*^2^(2) = 8.4, *p* = 0.0151). In TA, the attenuation slope was greater in young than older adults at 30% (*p* = 0.0003; *d* = 0.49) and 50% (*p* = 0.0011; *d* = 0.42) but not at 70% MVF (*p* = 0.9135; *d* = 0.01). Significant interaction contrasts from 30 to 70% (*p* = 0.0002; *d* = 0.48) and from 50 to 70% MVF (*p* = 0.0005; *d* = 0.41) also indicated that attenuation slope in young adults decreased relative to old with increasing force. In VL, young adults had a greater attenuation slope than older at 30% (*p* = 0.0075; *d* = 0.43), with no differences noted at 50% (*p* = 0.3025; *d* = 0.14) and a greater attenuation slope of older than young at 70% MVF (*p* = 0.0179; *d* = 0.35). Additionally, in VL, significant interaction contrasts from 30 to 50% (*p* = 0.0001; *d* = 0.57) and 30 to 70% MVF (*p* < 0.0001; *d* = 0.78) showed that attenuation slope of young adults decreased to a greater extent than older adults with increasing contraction force. In TA, both young and older adults’ attenuation slope decreased with increasing contraction level, from 30 to 50% (both *p* < 0.0001, both *d* ≥ 0.49) and 30 to 70% (young, *p* < 0.0001, *d* = 0.74; older, *p* = 0.0167, *d* = 0.27) but not 50 to 70% MVF (young, *p* = 0.2696, *d* = 0.19; older, *p* = 0.0624, *d* = 0.22). In VL with increasing contraction, young adults’ attenuation slope decreased (all *p* ≤ 0.0022; *d* ≥ 0.40), whereas older adults’ attenuation slope was unaffected by contraction level (30 to 50%, *p* = 1.0000, *d* = 0.02; 30 to 70%, *p* = 0.3111, *d* = 0.18; 50 to 70%, *p* = 0.0620; *d* = 0.19).

### Summary

The non-linearity of the ascending discharge rate (brace height) showed limited age-related differences, with older adults exhibiting greater values than young only at 30% MVF in VL. Acceleration slope was lower in older adults at all TA contraction levels but did not differ in VL, whereas attenuation slope was lower in older adults at lower TA contraction levels with mixed effects in VL. The modulation of these slopes across contraction levels was attenuated in older adults, with age-related differences overall more pronounced in TA than VL.

## Discussion

This study examined age-related differences in MU discharge rate modulation and estimates of PICs across a range of contraction levels and whether the magnitude of potential age-group differences varied between two lower limb muscles (TA and VL). Older adults exhibited lower knee extension and dorsiflexion isometric strength than young but with a greater age-related difference in knee extension than dorsiflexion strength. Muscle peak ACSA was lower only in the quadriceps of older adults, whereas it was greater in the shank anterior compartment of older adults compared to young adults. We observed that TA MU discharge rate was lower in older compared to young adults across all contraction levels. However, contrary to our hypothesis, the age-related differences in MU discharge rate were not exacerbated at higher contraction levels, and in the VL an age effect was only evident during low to moderate contractions (30% and 50% MVF). The onset-offset hysteresis (ΔF), an estimate of PIC contribution to self-sustained MU discharge, was lower at all contraction levels in older adults in TA but only lower at 70% MVF in VL. Interestingly, young adults demonstrated a steeper increase in ΔF across contraction levels compared to older adults. Conversely, ΔF normalised to maximum theoretical test unit hysteresis, was greater in older than young adults at 30% and 50% MVF in TA and at 30% MVF in VL. The geometric analyses of the ascending MU discharge patterns revealed the largest age-related differences at lower contraction levels, with the extent of non-linearity of the ascending MU discharge (brace height) shown to be greater in older compared to young adults at 30% MVF in VL. Similarly, attenuation slope, a supplemental measure of brace height, was lower in older adults during lower-level (TA 30 and 50%, VL 30% MVF), but not high-level (70% MVF) contractions. Finally, older adults had a lower initial MU discharge rate acceleration slope compared to young in TA across all contraction levels, but with no age-related differences in VL.

Collectively, these findings indicate that the magnitude of age-related alterations in strength, muscle size, and MU properties differs across muscles. The age-related weakness of the knee extensors appeared to be driven primarily by morphology, whereas the dorsiflexor weakness of older adults was more plausibly attributable to neural mechanisms given their greater muscle size, the details of which are discussed below.

### Motor unit discharge rate and neuromuscular contributions to ageing related strength differences

We found that MU discharge rate was lower in older adults compared with young in five out of six comparisons across muscles and contraction levels. Specifically, in the TA, discharge rate was lower in older adults compared with young at all contraction force levels tested (30, 50, and 70% MVF). Previous data in TA has yielded inconsistent outcomes, potentially due to small sample sizes in previous studies (e.g. *n* = 6–8 per group [8, 12]), low MU counts (~ 4–24 total MUs at each contraction level [12]) and the age of participants. For example, one investigation found a lower discharge rate in older adults (~ 82 years) at all force levels (10–100% MVF [8]), whereas another demonstrated age-related differences in MU discharge rate only during maximal, but not any submaximal force levels (≤ 60% MVF [12]) in older adults aged 70 years. Furthermore, McNeil et al. [57] demonstrated that age-related differences in MU discharge rate at submaximal force levels were only apparent when comparing very old (~ 82 years), but not older (~ 66 years) to young adults. Consistent with these findings, our results in a larger population (*n* = 30 per group) and a greater sample of TA motor units indicate that given sufficient age (≈73 years on average), older adults exhibit lower MU discharge rate across several submaximal forces compared to young, but the magnitude of this difference does not seem to be dependent on contraction force level.

In the VL, a lower discharge rate in older adults was only noted at 30% and 50%, but not 70% MVF. The finding of a lower MU discharge rate in older compared to young adults is generally in accordance with prior reports of low-force contractions in VL [11, 58], though no age-related differences have also been shown at some submaximal contraction levels (20–60% MVF [29, 58]). Despite no between-group differences in VL MU discharge rate at 70% MVF, we showed greater increases in VL MU discharge rate of younger adults with increasing contraction level, suggesting differences in motoneuron input–output gain control, potentially reflecting differences in the organisation of motor commands (for details, see below).

The pattern of discharge rate modulation across contraction levels in young and older adults differed between TA and VL as evidenced by both group × muscle and group × muscle × contraction level interactions, indicating greater age-related differences in TA than in VL. In contrast, the age-related differences in isometric strength were greater for the knee extensors than dorsiflexors, consistent with prior reports [11, 15]. This apparent dissociation suggests a muscle-specific contribution of neural and morphological factors to the age-related differences in strength. Specifically, older adults exhibited smaller quadriceps peak ACSA, consistent with several prior studies [59–61]. However, older adults had a larger shank anterior compartment compared to young which, whilst in contrast to a prior study finding no age-related differences [16], might be related to differences in non-contractile tissue [62] and/or the greater body mass of older adults in this study. Thus, the large difference in knee extension strength with age was primarily due to the substantial difference in quadriceps size and secondarily the smaller differences in MU discharge rate in VL. Conversely, for the dorsiflexors the more marked differences in MU discharge rate may have been somewhat countered by the greater anterior compartment size of older adults to produce a more modest age-related difference in dorsiflexor strength. We additionally note that the observations of the lower TA MU discharge rates of older adults are compatible with age-related MU remodelling, which has been shown to be more readily present in TA than in VL of pre-sarcopenic and non-sarcopenic older individuals [19]. This differential remodelling might have contributed to the differing relationship between MU discharge rate and force output between the two muscle groups. Altogether, these findings support the notion of a large heterogeneity and anatomical specificity in the neural and morphological underpinnings of the age-related differences in muscle strength.

### Possible mechanisms underlying age-related differences in motor unit discharge patterns across contraction levels

Our results showed that the discharge rate hysteresis of MUs (ΔF) was lower in older adults compared to young at all force levels in TA, but only at 70% MVF in VL. Previous studies comparing young and older adults have only examined 20% MVF contractions, but are consistent with our data in TA, demonstrating lower ΔF in older adults in TA and soleus [28], biceps and triceps brachii [30], and VL (males only [29]). The present study extends prior observations by examining *tracked* MUs in a large sample of young and older males and females performing isometric contractions up to 70% MVF. Interestingly, in both muscles, young adults showed a greater increase in ΔF with increasing contraction level compared to older adults, which corresponded with a greater gain in MU discharge rates with contraction level in VL but not in TA. This age-related attenuation in the modulation of discharge rate hysteresis with greater contraction forces is in agreement with baseline data from healthy older adults in an intervention study (≤ 40% MVF [63]) and a recent study in sarcopenic older adults (≤ 60% MVF [64]). Previously, the age-related differences in MU discharge rate hysteresis have largely been interpreted as a smaller PIC contribution to MU discharge rate in older compared to young adults [28, 30] due to the role of PIC in prolongation of motoneuron discharge [22, 23], mediated either via monoaminergic inputs or altered inhibitory PIC control in older adults. There is some evidence for this in our data as acceleration slopes, likely indicative of PIC-related acceleration in discharge rates, were also found to be greater in young adults in TA across all contraction levels. The initiation and initial acceleration of motoneuron discharge is facilitated by fast-acting Na^+^ PICs which are facilitated by serotonin and noradrenaline [65]. Although the mechanisms at individual channel level cannot be inferred directly from extracellular MU recordings, the ectopic expression of Na_V_1.8 channels and resultant alteration of membrane excitability documented in motor axons of aged mice [66] raises the possibility that dysfunction of Na^+^ PICs could contribute to the lower MU discharge rate acceleration in older TA. The consistently lower acceleration slopes across all contraction levels in TA, taken together with lower ∆F, are nonetheless consistent with a reduced contribution of PIC-mediated amplification to MU discharge rate of older adults.

Nevertheless, when accounting for discharge rate modulation of the control unit, which provides a proxy of synaptic input for the ΔF calculation (i.e. normalised ΔF), discharge rate hysteresis in older adults was greater in older adults than young at 30% and 50% MVF in TA and 30% in VL. This was also accompanied by the lack of age-related differences in discharge rate non-linearity, which, in in silico studies, has been linked to the relative contribution of PICs to the ascending discharge rate modulation mediated by monoaminergic inputs [31, 50, 51]. Taken together, the blunted modulation of normalised ∆F across contraction level in aged adults is consistent with insufficient excitation-inhibition coupling for PIC facilitation of MU discharge rates rather than altered neuromodulatory drive. This pattern appears to be less pronounced in VL where greater brace height and normalised ΔF values in older adults at 30% MVF suggest a comparatively preserved relative PIC contribution to MU discharge rate at low forces, potentially contributing to the smaller age-related differences in VL MU discharge rate. Examination of MU discharge pattern metrics further supports this interpretation. In TA, older adults exhibited lower acceleration slopes, consistent with reduced amplification of the ascending discharge rates, alongside lower attenuation slopes at low to moderate contraction levels, which has been linked to a more proportional/less reciprocal (i.e. push–pull) pattern of excitation-inhibitory coupling in simulations [50]. This more proportional excitation-inhibition pattern of older adults with greater contraction forces, might reflect either blunted upregulation of excitatory and/or increased inhibitory input, potentially resulting in reduced net synaptic input. In contrast, in VL, no between-group differences were noted in acceleration slopes, suggesting relatively preserved PIC acceleration of MU discharge rate. However, unlike young adults, older adults did not modulate their attenuation slope with contraction level, which might reflect less dynamic inhibitory control, consistent with prior observation at low contraction levels [27]. Together, the muscle × age group × contraction level interactions observed in our discharge pattern metrics suggests that ageing differentially affects the force-dependent regulation of excitation-inhibition coupling.

Although the precise nature of age-related alterations in net synaptic input as a result of altered excitation-inhibition coupling cannot be determined from the present data directly, both alterations in excitatory [67, 68] and inhibitory [69, 70] synaptic inputs have been demonstrated in ageing adults. Further support for potential alterations in excitation-inhibition coupling comes from a post-mortem analysis of human spinal motoneurons which indicated that older adults exhibit a reduced ratio of excitatory to inhibitory synapses, largely due to shedding of excitatory synaptic inputs from soma and dendritic tree [71]. Whatever the precise nature of alterations in synaptic input, the more proportional excitatory-inhibitory synaptic coupling of older adults with greater contraction forces is a plausible mediator of their blunted modulation of discharge rate hysteresis, which might contribute to their lower MU discharge rate and voluntary maximal force producing capacity.

### Potential functional implications

The differing contributions of neural and morphological factors to age-related strength differences across the two examined muscles have potential implications for both the interpretation of strength as an indicator of neuromuscular ageing and the design of interventions targeting the age-related decline in physical function. The relatively modest differences in dorsiflexor strength in older adults, but consistent differences in neural properties, suggest that modest or absent functional differences in isometric strength might, in some muscle groups, be a poor indicator of ageing. Whether the muscle-specific patterns we report reflect different stages along a common ageing trajectory or inherent differences between motor pools cannot be determined from the present cross-sectional data in a non-sarcopenic cohort. Replication in sarcopenic and frail populations, along with longitudinal data, is required to characterise how the relative contributions of neural and morphological factors evolve with functional changes [64]. Such future studies, along with the evidence presented herein, are also critical to inform whether interventions are most effectively tailored to a given muscle group—for example, resistance training and nutritional strategies that prioritise morphological versus neural adaptations depending on the predominant contributor to the age-related weakness. In this regard, resistance training has been shown to be a potent stimulus for increases in neural drive (including PIC-mediated processes [63, 72]) as well as muscle hypertrophy of older adults [73]. Furthermore, pharmacological interventions, particularly in populations in whom exercise is not feasible, might be possible, both for increasing motoneuron excitability [74] and preventing the loss of muscle size [75], although the extent to which their actions could be effectively localised remains to be explored.

### Limitations

The present study has numerous strengths including a large, mixed sample of both males and females, a high yield of identified MUs that were matched across contraction levels allowing for the comparison of MUs receiving the same synaptic input without duplication, measurements of both a greater and less preserved muscle with ageing, and the use of ‘gold-standard’ MRI to measure muscle size. Nevertheless, there are some limitations that are worthy of consideration. First, physical activity levels were only estimated using a self-report questionnaire at one time point and it is unknown to what extent lifelong physical activity habits, particularly of individuals in the older group, could contribute to their neuromuscular function. However, a recent study showed that discharge rate hysteresis appears not to differ between non-sarcopenic older individuals (such as those in our study) and highly active older adults [64], suggesting the potential heterogeneity in physical activity differences in our sample had limited influence on results. Second, although the present study was the first to investigate age-related differences in discharge rate hysteresis and ascending discharge linearity in *tracked* MUs at high forces (up to 70% MVF), we were unable to assess this during maximal contractions as the rate of force increase in these contraction types could not be controlled. Further research and methodological advances are required to understand the contribution of alterations in excitatory, inhibitory and neuromodulatory inputs to the age-related loss in maximal force and rate of force development. Third, we investigated MU discharge characteristics of two muscles (TA and VL) and showed that the age-related differences in MU discharge properties appear to be muscle and muscle × contraction level dependent. Although this is consistent with prior suggestions [7], the results cannot necessarily be extrapolated to other muscle groups. Further, from the perspective of explaining age-related differences in strength, whilst tibialis anterior contributes to a large proportion of dorsiflexion torque (~ 70% [76]), vastus lateralis is only one of the four quadriceps muscles that contributes to the knee extension torque. Nevertheless, prior studies have shown similar results when comparing MU discharge properties in young and older adults in different quadriceps muscles [77]. Fourth, we also did not measure antagonist muscle activity; therefore, it cannot be determined to what extent differences in antagonist co-activation between age groups and joint actions may have influenced the results. Fifth, as already acknowledged in the Methods, decomposition of HDsEMG is inherently biased toward higher-threshold units with larger surface potentials [37], which might differentially affect the population sampled in young versus older adults given age-related changes in MU size and territories, not least due to the reported MU remodelling in ageing populations [19, 57]. Nevertheless, similar (TA) or even greater number of the identified units (VL) in older compared to young adults suggests we reported the activity of representative samples of MUs. Finally, though we incorporated sex as a covariate in our statistical model, sex differences were not investigated directly in this study as they did not form part of the research questions, nor was the sample appropriately powered for an additional independent variable and the ensuing 4-way interaction (age group × contraction level × muscle × sex). As such, extrapolation of results to either sex should be done with caution. Sex differences in MU discharge properties have been shown previously in both young [46] and older [78] individuals, and we intend to explore the comparison of MU discharge properties and the underlying mechanisms between young and older adults in relation to sex in future studies.

## Conclusions

In summary, young and older adults exhibit distinct, muscle-specific patterns of MU discharge rate modulation across contraction levels. These observations can be situated within the wider biology of neuromuscular ageing in which altered synaptic input, intrinsic motoneuron properties, and motor unit remodelling have all been proposed to contribute to age-related changes in PIC-mediated MU discharge [79]. Older adults showed blunted discharge rate modulation across contraction levels and reduced discharge hysteresis, particularly in TA, indicating a diminished capacity to amplify synaptic input, likely due to altered excitation–inhibition coupling. In contrast, neural differences in VL emerged only at specific contraction levels, indicating that the smaller quadriceps muscle size in older adults is likely the primary contributor to the lower quadriceps maximal force. Leveraging a large mixed-sex cohort, a high yield of tracked MUs across contraction levels, and gold-standard MRI measurements of muscle size, the present study therefore provides robust evidence that the ageing process alters neuromuscular function in a muscle-specific manner. Whilst neural constraints might predominate in the dorsiflexors, morphological alterations likely played a greater role in knee extensors, underscoring the heterogeneous anatomically specific nature of neuromuscular ageing.

## Supplementary Information

Below is the link to the electronic supplementary material.

ESM 1 (PDF 145 KB)

ESM 2 (PDF 141 KB)

ESM 3 (DOCX 22.8 KB)
