# Supplementary material for: Age-related differences in motor unit discharge rate modulation across contraction levels
Source: GeroScience. 2026 Jun 30;48(6):7575–96. doi: 10.1007/s11357-026-02383-9 (PMC13638850; doi:10.1007/s11357-026-02383-9)
Supplement: Supplementary file 1 [file 11357_2026_2383_MOESM1_ESM.pdf]

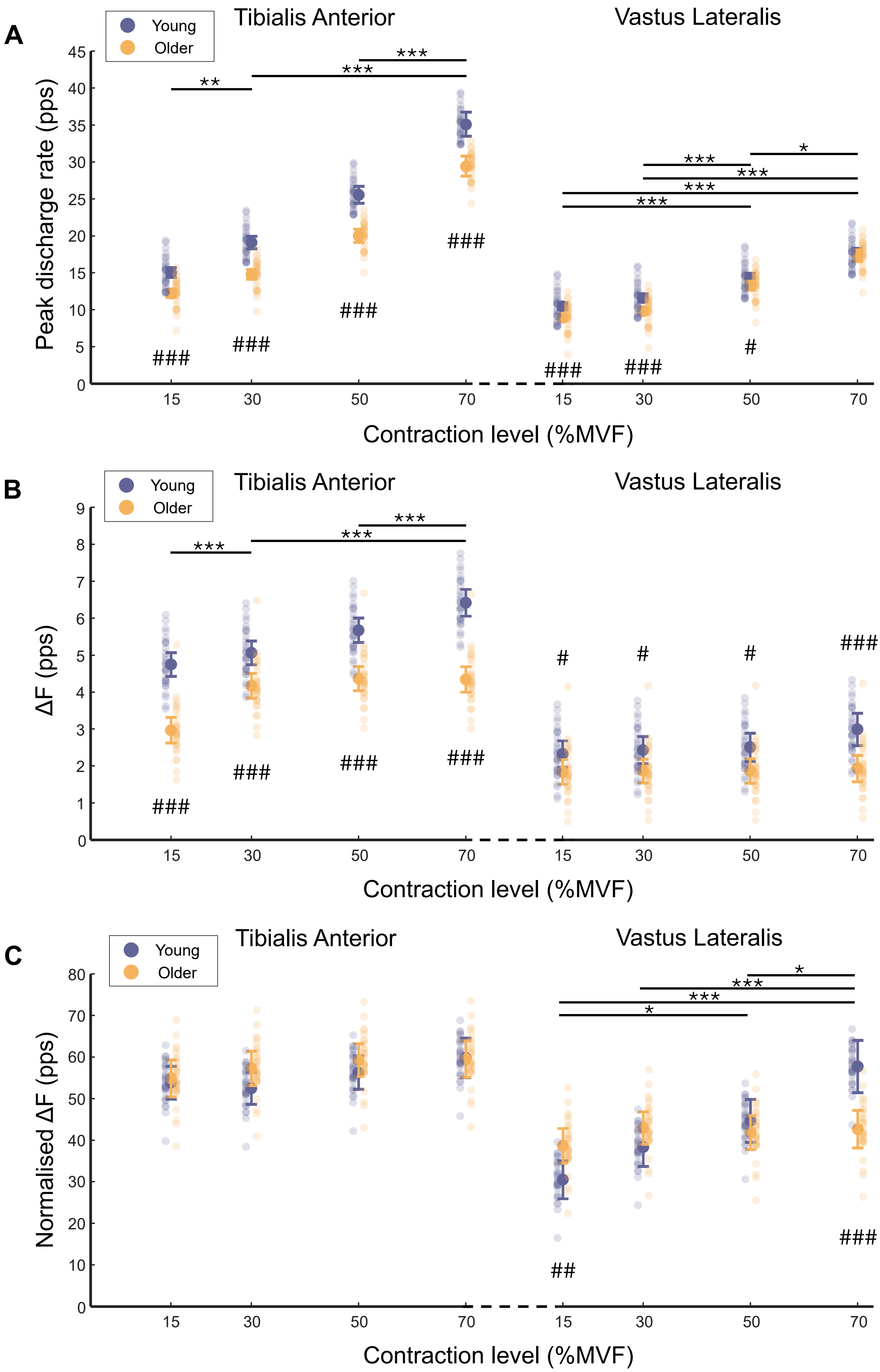

A
Young
Older
Tibialis Anterior
Vastus Lateralis
Peak discharge rate (pps)
45
40
35
30
25
20
15
10
5
0
**
***
***
***
***
***
***
*
###
###
###
###
###
###
#
15
30
50
70
15
30
50
70
Contraction level (%MVF)
B
Young
Older
Tibialis Anterior
Vastus Lateralis
ΔF (pps)
9
8
7
6
5
4
3
2
1
0
***
***
***
###
###
###
###
#
#
#
###
15
30
50
70
15
30
50
70
Contraction level (%MVF)
C
Young
Older
Tibialis Anterior
Vastus Lateralis
Normalised ΔF (pps)
80
70
60
50
40
30
20
10
0
*
***
***
*
##
###
15
30
50
70
15
30
50
70
Contraction level (%MVF)
