# Supplementary material for: Age-related differences in motor unit discharge rate modulation across contraction levels
Source: GeroScience. 2026 Jun 30;48(6):7575–96. doi: 10.1007/s11357-026-02383-9 (PMC13638850; doi:10.1007/s11357-026-02383-9)
Supplement: Supplementary file 2 [file 11357_2026_2383_MOESM2_ESM.pdf]

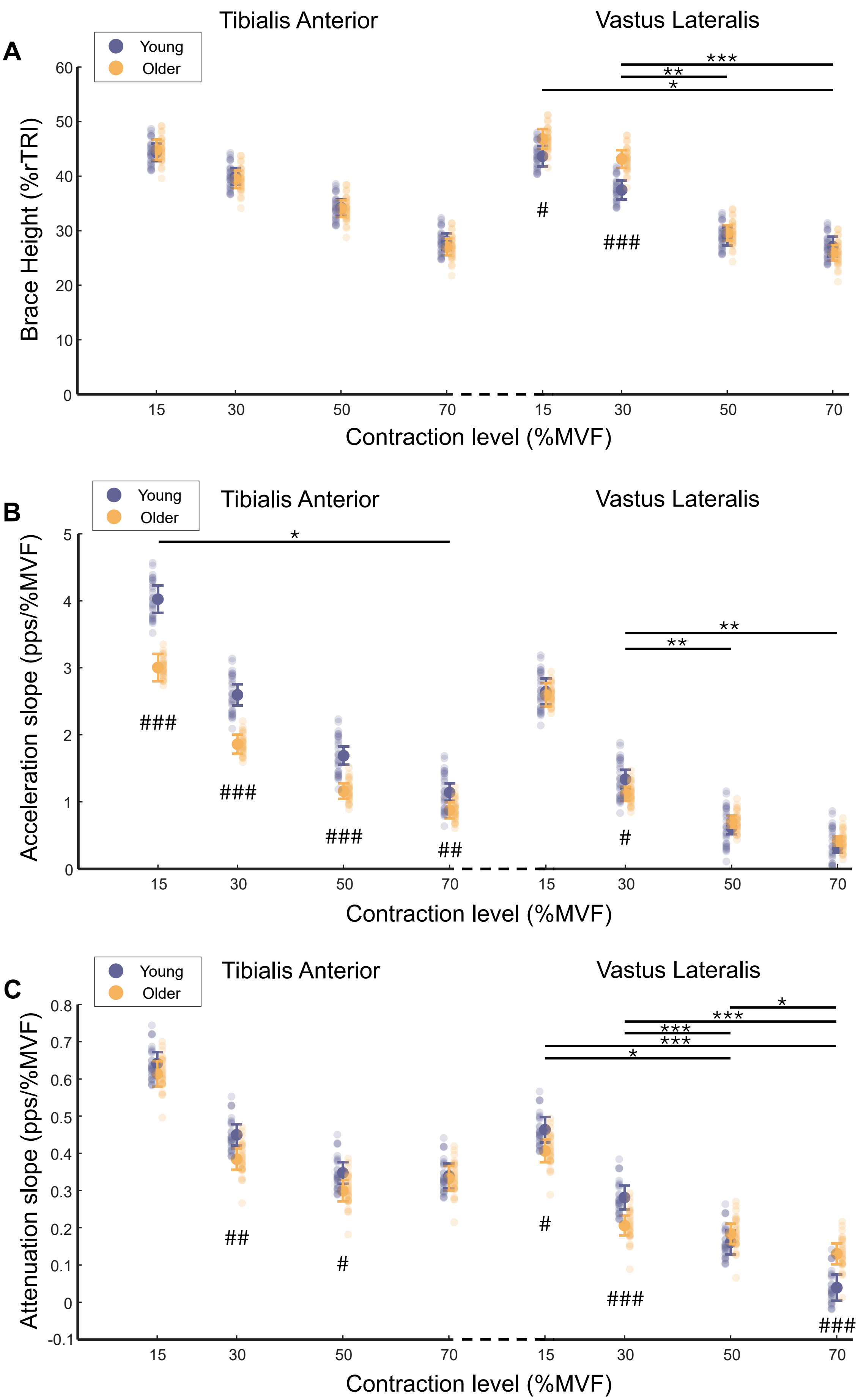
A
Tibialis Anterior
Vastus Lateralis
Young
Older
Brace Height (%rTRI)
0
10
20
30
40
50
60
***
**
*
#
###
15
30
50
70
15
30
50
70
Contraction level (%MVF)
B
Tibialis Anterior
Vastus Lateralis
Young
Older
Acceleration slope (pps/%MVF)
0
1
2
3
4
5
*
**
**
###
###
###
##
#
15
30
50
70
15
30
50
70
Contraction level (%MVF)
C
Tibialis Anterior
Vastus Lateralis
Young
Older
Attenuation slope (pps/%MVF)
-0.1
0
0.1
0.2
0.3
0.4
0.5
0.6
0.7
0.8
*
***
***
***
*
##
#
#
###
###
15
30
50
70
15
30
50
70
Contraction level (%MVF)
