# Supplementary material for: Age-related differences in motor unit discharge rate modulation across contraction levels
Source: GeroScience. 2026 Jun 30;48(6):7575–96. doi: 10.1007/s11357-026-02383-9 (PMC13638850; doi:10.1007/s11357-026-02383-9)
Supplement: Supplementary file 3 [file 11357_2026_2383_MOESM3_ESM.docx]

**Supplementary Material**

**Supplemental Table 1.** Number of identified tracked motor units.

|  |  | **Young** | **Older** |
| --- | --- | --- | --- |
| **Tibialis Anterior** | | | |
| **30-50% MVF** | **Total**  **Mean ± SD** | **253**  **8 ± 6** | **339**  **11 ± 6** |
| **50-70% MVF** | **Total**  **Mean ± SD** | **247**  **8 ± 6** | **342**  **11 ± 6*** |
| **30-50-70% MVF** | **Total**  **Mean ± SD** | **111**  **4 ± 3** | **209**  **7 ± 5**** |
| **Unique** | **Total**  **Mean ± SD** | **388**  **13 ± 8** | **474**  **16 ± 7** |
| **Vastus Lateralis** | | | |
| **30-50% MVF** | **Total**  **Mean ± SD** | **107**  **4 ± 4** | **285**  **10 ± 7***** |
| **50-70% MVF** | **Total**  **Mean ± SD** | **107**  **4 ± 4** | **297**  **10 ± 9**** |
| **30-50-70% MVF** | **Total**  **Mean ± SD** | **35**  **1 ± 2** | **187**  **6 ± 6***** |
| **Unique** | **Total**  **Mean ± SD** | **179**  **6 ± 5** | **394**  **13 ± 11**** |

N.b. The values presented are both total and mean ± standard deviation (SD) motor units identified and tracked across 30-50%, 50-70% and 30-50-70% MVF, also presented is the number of unique motor units identified. MVF, maximum voluntary force. ***p < 0.001, **p < 0.01, *p < 0.05, denotes statistically significant difference to young adults.

**Results of Non-Tracked Motor Units**

The total number of MUs recorded from TA was 1895 and 1870 (p = 0.9084) in older adults and young adults, respectively. In VL older adults had a greater number of MUs identified compared to young adults (1634 vs. 971, p = 0.0094; Supplemental Table 2).

**Supplemental Table 2.** Number of identified non-tracked motor units.

|  |  | **Young** | **Older** |
| --- | --- | --- | --- |
| **Tibialis Anterior** | | | |
| **15% MVF** | **Total**  **Mean ± SD** | **561**  **19 ± 8** | **467**  **16 ± 6** |
| **30% MVF** | **Total**  **Mean ± SD** | **492**  **16 ± 8** | **492**  **16 ± 6** |
| **50% MVF** | **Total**  **Mean ± SD** | **435**  **15 ± 7** | **497**  **17 ± 8** |
| **70% MVF** | **Total**  **Mean ± SD** | **382**  **13 ± 7** | **439**  **15 ± 7** |
| **Vastus Lateralis** | | | |
| **15% MVF** | **Total**  **Mean ± SD** | **295**  **10 ± 5** | **424**  **14 ± 9*** |
| **30% MVF** | **Total**  **Mean ± SD** | **269**  **9 ± 6** | **456**  **15 ± 11**** |
| **50% MVF** | **Total**  **Mean ± SD** | **225**  **8 ± 6** | **410**  **14 ± 11**** |
| **70% MVF** | **Total**  **Mean ± SD** | **182**  **7 ± 5** | **344**  **13 ± 10*** |

N.b. The values given for both total and mean ± standard deviation (SD) are for unique motor units identified across the two contractions at each relative contraction force. MVF, maximum voluntary force. **p < 0.01, *p < 0.05, denotes statistically significant difference to young adults.

Peak discharge rate during triangular contractions had main effects of age group (χ^2^(1) = 36.5, p < 0.0001), muscle (χ^2^(1) = 9866.3, p < 0.0001) and contraction level (χ^2^(3) = 9851.3, p < 0.0001; Supplemental Fig 1A). There were also interactions of age group × muscle (χ^2^(1) = 150.9, p < 0.0001), age group × contraction level (χ^2^(3) = 74.9, p < 0.0001), muscle × contraction level (χ^2^(3) = 443.7, p < 0.0001) and age group × muscle × contraction level (χ^2^(3) = 34.1, p < 0.0001). In the TA, young adults had a greater discharge rate than older adults at each contraction level (all p < 0.0001; all *d* ≥ 2.95), with the discharge rate increasing across contraction levels to a greater extent in young compared to older from 15 to 30% MVF (p = 0.0067; *d* = 0.86) but with the inverse of this occurring from 30 to 70% and from 50 to 70% MVF (both p ≤ 0.0002; both *d* ≥ 1.16). In the VL, young adults had a greater discharge rate at 15%, 30% and 50% but not at 70% MVF compared to older adults (15%, p < 0.0001, *d* = 2.64; 30%, p < 0.0001, *d* = 2.79; 50%, p = 0.0346, *d* = 1.19; 70%, p = 0.8095, *d* = 0.14). There was also a different pattern of discharge rate modulation between young and older, with older adults’ discharge rate increasing to a greater extent than young from 15 to 50%, 15 to 70%, 30 to 50%, 30 to 70% (all p < 0.0001; all *d* ≥ 1.45) and from 50 to 70% MVF (p = 0.0182; *d* = 1.06). In both groups and muscles, with increasing contraction level peak discharge rate increased (all p < 0.0001; all *d* ≥ 1.56)

The discharge rate hysteresis of MUs (ΔF), showed main effects of age group (χ^2^(1) = 34.6, p < 0.0001), muscle (χ^2^(1) = 1961.8, p < 0.0001) and contraction level (χ^2^(3) = 61.0, p < 0.0001; Supplemental Fig 1B). There were also interactions of age group × muscle (χ^2^(1) = 54.1, p < 0.0001), age group × contraction level (χ^2^(3) = 36.2, p = 0.0001), muscle × contraction level (χ^2^(3) = 72.9, p = 0.0001) and age group × muscle × contraction level (χ^2^(3) = 12.5, p = 0.0059). In the TA, young adults had greater ΔF than older adults at all contraction levels (all p ≤ 0.0002, all *d* ≥ 0.87). There was also a significantly larger increase in ΔF of older adults compared to young from 15 to 30% MVF (p < 0.0001; *d* = 0.86) and, on the contrary, a significantly larger increase in young compared to older adults from 30 to 70% (p < 0.0001; *d* = 1.15) and from 50 to 70% MVF (p = 0.0004; *d* = 0.75), indicating that group differences were more pronounced at the lowest and highest contraction levels. In VL, young adults had a greater ΔF than older at all contraction levels (15%, p = 0.0475, *d* = 0.47; 30%, p = 0.0225, *d* = 0.55; 50%, p = 0.0127, *d* = 0.62; 70%, p = 0.0002, *d* = 1.03). In VL of older adults, across contraction levels there was no significant difference in ΔF (all p = 1.0000; all *d* ≤ 0.09). Similarly, in VL of young adults, across contraction levels there was no significant difference in ΔF (all p ≥ 0.0710; all *d* ≤ 0.65). In TA of young adults, from 15 to 30% MVF (p = 0.3970; *d* = 0.31) there was no significant difference in ΔF, however, across all other comparisons with increasing contraction level ΔF increased (all p ≤ 0.0002; all *d* ≥ 0.59). In TA, of older adults despite an increase in ΔF from 15% to 30%, 15% to 50%, and 15% to 70% MVF (all p < 0.0001; all *d* ≥ 1.17), there was no difference between contraction levels from 30% to 50%, 30% to 70%, and 50% to 70% MVF (all p = 1.0000; all *d* ≤ 0.19).

For normalised ΔF, there was no significant main effects of age group (χ^2^(1) = 0.9, p = 0.3517), but significant main effects were noted for muscle (χ^2^(1) = 289.9, p < 0.0001) and contraction level (χ^2^(3) = 11.8, p = 0.0080; Supplemental Fig 1B). There were also interactions of age group × contraction level (χ^2^(3) = 20.4, p = 0.0001), muscle × contraction level (χ^2^(3) = 12.4, p = 0.0061) and age group × muscle × contraction level (χ^2^(3) = 19.1, p = 0.0003), but not for age group × muscle (χ^2^(1) = 54.1, p < 0.0001). In TA, there were no between group differences at any contraction level (all p ≥ 0.0906; all *d* ≤ 0.15), and no between group interactions across contraction levels. In VL, older adults had a greater normalised ΔF than young at 15% (p = 0.0080; *d* = 0.26), with no differences at 30% or 50% (both p ≥ 1403; both *d* ≤ 0.15), but at 70% MVF (p = 0.0001; *d* = 0.48) young adults had a greater normalised ΔF than older adults. In VL, there were interactions from 15% to 50%, 15% to 70%, 30% to 70%, and 50% to 70% MVF (all p ≤ 0.0182; all *d* ≥ 0.35) showing that with increasing contraction level young adults normalised ΔF increased relative to older adults. In TA, there was no difference in normalised ΔF across contraction levels in older adults (all p = 1.0000; all *d* ≤ 0.15) or young adults (all p ≥ 0.1305; all *d* ≤ 0.23). In VL, there were also no differences in older adults normalised ΔF across contraction levels (all p = 1.0000; all *d* ≤ 0.14). Whereas in young adults VL, normalised ΔF increased from 15% to 50%, 15% to 70%, 30% to 70%, and 50% to 70% MVF (all p ≤ 0.0048; all *d* ≥ 0.42).

**Supplemental Figure 1. Motor unit discharge rate and hysteresis of non-tracked motor units in tibialis anterior and vastus lateralis of young and older adults by contraction level.** A: Peak discharge rate (pulses per second, pps). B: onset-offset discharge hysteresis of MU pairs (ΔF). C: ΔF normalised to the difference between control unit discharge rate at test unit recruitment and at control unit derecruitment. Blue (young) and orange (older) circles represent estimated marginal means from the linear mixed effects statistical model, with the error bars representing 95% confidence intervals. Transparent circles represent individual participant model adjusted means for the whole statistical model (i.e. not by muscle). ^###^p < 0.001, ^##^p < 0.01, ^#^p < 0.05 denotes between group differences at each contraction level, ^***^p < 0.001, ^**^p < 0.01, ^*^p < 0.05 denotes interaction contrast between groups across contraction levels. MVF = maximal voluntary force.

The ascending discharge rate non-linearity (brace height) showed no main effect of age group (χ^2^(1) = 1.1, p = 0.3005) but main effects were noted for muscle (χ^2^(1) = 9.7, p = 0.0019) and contraction level (χ^2^(3) = 833.5, p < 0.0001; Supplemental Fig 2A). Interaction effects were noted for each of age group × muscle (χ2(1) = 14.7, p = 0.0001), age group × contraction level (χ^2^(3) = 14.2, p = 0.0026), muscle × contraction level (χ^2^(3) = 57.8, p < 0.0001) and age group × muscle × contraction level (χ^2^(3) = 12.9, p = 0.0049). In either muscle, the only between group differences noted were at 15% (p = 0.0142; *d* = 0.28) and 30% MVF (p < 0.0001; *d* = 0.53) in VL where older adults had a greater brace height than young. There were also interaction contrasts in VL such that brace height in older adults decreased to a greater extent from 15 to 70% (p = 0.0190; *d* = 0.41), 30 to 50% (p = 0.0030; *d* = 0.46) and from 30 to 70% MVF compared to young adults (p < 0.0001; *d* = 0.66). In both groups for each muscle, as contraction level increased brace height decreased (all p ≤ 0.0017; *d* ≥ 0.32), except for 50% to 70% MVF in young adults VL (p = 1.0000; *d* = 0.21).

For the acceleration slope, there were main effects of age group (χ^2^(1) = 27.8, p < 0.0001), muscle (χ^2^(1) = 837.8, p < 0.0001) and contraction level (χ^2^(3) = 1755.7, p < 0.0001; Supplemental Fig 2B). There were also interaction effects for age group × muscle (χ^2^(1) = 89.5, p < 0.0001), age group × contraction level (χ^2^(3) = 20.3, p = 0.0001) and muscle × contraction level (χ^2^(3) = 27.5, p < 0.0001) but not for age group × muscle × contraction level (χ^2^(3) = 5.0, p = 0.1719). In TA, the acceleration slope was greater in young than older adults at all contraction levels (15%, 30%, 50%, all p < 0.0001, all d ≥ 0.57; and 70% p = 0.0018, *d* = 0.34). In VL, young adults had a greater acceleration slope than older adults at 30% MVF (p = 0.0144; *d* = 0.25) with no differences at 15%, 50%, or 70% MVF (15%, p = 0.6919, *d* = 0.04; 50%, p = 0.1953, *d* = 0.14; 70%, p = 0.0890, *d* = 0.20). In TA, young adults acceleration slope decreased to a greater extent than older adults from 15 to 70% MVF (p = 0.0178; *d* = 0.35) and in VL the same interaction contrast was noted from 30 to 50% (p = 0.0077; *d* = 0.39) and 30 to 70% MVF (p = 0.0029; *d* = 0.45). With increasing contraction level, acceleration slope decreased in both muscles in young (all p < 0.0001; *d* ≥ 0.56) and older adults (all p ≤ 0.0018; all *d* ≥ 0.36).

Attenuation slope showed main effects of age group (χ^2^(1) = 4.0, p = 0.0463), muscle (χ^2^(1) = 907.2, p < 0.0001) and contraction level (χ^2^(3) = 894.9, p < 0.0001; Supplemental Fig 2C). Significant interactions were observed for age group × muscle (χ^2^(1) = 4.4, p = 0.0369), age group × contraction level (χ^2^(3) = 41.0, p < 0.0001), muscle × contraction level (χ^2^(3) = 37.6, p < 0.0001) and age group × muscle × contraction level (χ^2^(3) = 21.3, p < 0.0001). In TA, the attenuation slope was greater in young than older adults only at 30% (p = 0.0013; *d* = 0.35) and 50% MVF (p = 0.0213; *d* = 0.26). In the VL, young adults had a greater attenuation slope than older at 15% (p = 0.0123; *d* = 0.30) and 30% (p = 0.0005; *d* = 0.42), with no differences noted 50% (p = 0.2868; *d* = 0.13), however, at 70% older adults had a greater attenuation slope than young (p < 0.0001; *d* = 0.55). Furthermore, in VL, there were interaction contrasts from 15% to 50% (p = 0.0109; *d* = 0.43), 15% to 70% (p < 0.0001; *d* = 0.85), 30% to 50% (p = 0.0004; *d* = 0.56), 30% to 70% (p < 0.0001; *d* = 0.97) and from 50% to 70% MVF (p = 0.0316; *d* = 0.41) all showing that young adults attenuation slope decreases to a greater extent than older adults with increasing contraction level. In TA, with increasing contraction level from 15% to 30% (young: p < 0.0001, *d* = 0.97; older: p < 0.0001, *d* = 1.18), 15% to 50% (young: p < 0.0001, *d* = 1.51; older: p < 0.0001, *d* = 1.64), 15 to 70% (young: p < 0.0001, *d* = 1.56; older: p < 0.0001, *d* = 1.46), and 30% to 50% (young: p < 0.0001, *d* = 0.54; older: p < 0.0001, *d* = 0.46) both groups attenuation slope decreased. From 30% to 70% (young: p < 0.0001, *d* = 0.59; older: p = 0.2426, *d* = 0.28) there was a decrease in young, but not in older adults and no differences were observed in either group from 50% to 70% MVF (young: p = 1.0000, *d* = 0.05; older: p = 1.0000, *d* = 0.18). In VL, young adults attenuation slope decreased with increasing contraction level (all p < 0.0001; all *d* ≥ 0.69), as did older adults (all p ≤ 0.0111; all *d* ≥ 0.31), except from 30% to 50% MVF (p = 1.0000; *d* = 0.13)

**Supplemental Figure 2. Motor unit discharge patterns of non-tracked motor units in tibialis anterior and vastus lateralis of young and older adults by contraction level.** A: The non-linearity of the ascending discharge rate (brace height, expressed as a percentage of right triangle, %rTRI). B: Acceleration slope of the ascending discharge (secondary discharge range; in pulses per second relative to maximal voluntary force, MVF). C: Attenuation slope of the ascending discharge rate (tertiary discharge range). Blue (young) and orange (older) circles represent estimated marginal means from the linear mixed effects statistical model, with the error bars representing 95% confidence intervals. Transparent circles represent individual participant model adjusted means for the whole statistical model (i.e. not by muscle). ^###^p < 0.001, ^##^p < 0.01, ^#^p < 0.05 denotes between group differences at each contraction level, ^***^p < 0.001, ^**^p < 0.01, ^*^p < 0.05 denotes interaction contrast between groups across contraction levels. MVF = maximal voluntary force.
